# Functions of Liver Natural Killer Cells Are Dependent on the Severity of Liver Inflammation and Fibrosis in Chronic Hepatitis C

**DOI:** 10.1371/journal.pone.0095614

**Published:** 2014-04-23

**Authors:** Emilie Fugier, Hélène Marche, Marie-Ange Thélu, Zuzana Macek Jílková, Nicolas Van Campenhout, Tania Dufeu-Duchesne, Vincent Leroy, Jean-Pierre Zarski, Nathalie Sturm, Patrice N. Marche, Evelyne Jouvin-Marche

**Affiliations:** 1 INSERM, U823, Grenoble, France; 2 Université Joseph Fourier, UMR-S823, Grenoble, France; 3 CHU-Grenoble, Clinique Universitaire d’Hépato-Gastroentérologie, Grenoble, France; 4 CHU-Grenoble, Département d’Anatomie et de Cytologie Pathologiques, Grenoble, France; Inserm, U1052, UMR 5286, France

## Abstract

During chronic hepatitis C virus (HCV) infection, the role of intra-hepatic (IH) natural killer (NK) cells is still controversial. To clarify their functions, we investigated anti-viral and cytotoxic activity of NK cells in human fresh liver biopsies. We compared the functions of IH-NK cells in HCV-infected and NASH patients in physiological conditions as well as after stimulation using flow cytometric and immunohistochemical analyses. Interestingly, few IH-NK cells produced anti-viral cytokine IFN-γ in HCV-infected patients similarly as in non-infected individuals. Spontaneous degranulation activity was extremely low in peripheral NK cells compared to IH-NK cells, and was significantly higher in IH-NK cells from HCV-infected patients compared to non-infected individuals. Immunohistochemical analysis revealed that perforin granules were polarized at the apical pole of IH-NK cells. The presence of CD107a and perforin in IH-NK cells demonstrated that NK cells exerted a cytolytic activity at the site of infection. Importantly, IH-NK cell functions from HCV-infected patients were inducible by specific exogenous stimulations. Upon *ex vivo* K562 target cell stimulations, the number of degranulating NK cells was significantly increased in the pool of IH-NK cells compared to circulating NK cells. Interestingly, after stimulation, the frequency of IFN-γ-producing IH-NK cells in HCV-infected patients was significantly higher at early stage of inflammation whereas the spontaneous IH-NK cell degranulation activity was significantly impaired in patients with highest inflammation and fibrosis Metavir scores. Our study highlights that some IH-NK cells in HCV-infected patients are able to produce INF-γ and degranulate and that those two activities depend on liver environment including the severity of liver injury. Thus, we conclude that critical roles of IH-NK cells have to be taken into account in the course of the liver pathogenesis associated to chronic HCV infection.

## Introduction

A large majority of hepatitis C virus (HCV)-infected patients develops a chronic disease with increasing hepatic injury over time [1], [2]. Despite intensive investigations, the phenomenon of persistent infection and parameters involved in tissue damage are not fully understood. Not surprisingly, NK cells, as one of the major components of the innate immune system, have been known to play an important role in the control of viral hepatitis for many years. Importantly, NK cells do not require priming to recognize infected cells and in addition, they contribute to T cell activation. Functional mechanisms of NK cells include: i) secretion of interferon-γ (IFN-γ), which has an antiviral effect and participates in the induction of the adaptive immune response; ii) a direct cytotoxicity to target cells via the degranulation of cytotoxic granules (perforin, granzyme); iii) and the induction of target cells apoptosis via the up-regulation of Fas ligand and tumor necrosis-related apoptosis-inducing ligand on the surface of NK cells [3], [4]. NK cell regulation depends on a fine balance between inhibitory and activating receptors which belong either to Immunoglobulin-like superfamily (Killer cell Immunoglobulin-like receptor or KIR), or to natural cytotoxicity receptors [4], [5] that are described as activating receptor able to recognize viral determinants [6], [7].

Intra-hepatic (IH)-NK cell functions are strongly influenced by liver microenvironment and are therefore modified depending on liver disease pathogenesis. In HCV-infected patients, IH-NK cells interact with the virus and the pool of these cells decrease with the severity of liver damages [8]–[10]. It has been reported that phenotypical features of NK cell subset correlate with clinical parameters scoring the evolution of HCV infection disease. Bonorino et al. [11] found a positive correlation between NKG2A^+^NK cells and the necro-inflammatory activity or fibrosis stage according to the Metavir scoring system. The study by Kramer et al. [12] demonstrated that NKp46^+^high IH-NK cell subset was inversely correlated with fibrosis stage, supporting the hypothesis that NK cells can play an important anti-fibrotic role due to the NK killing activity of hepatic stellate cells [13]. Nevertheless, a recent study suggests that IH-NK cell cytotoxic function is impaired in patients with chronic HCV infection [14] whereas another study provides evidence that IH-NK cells can be further activated by IFN-α antiviral therapy during HCV infection [15]. Thus, further studies are required to clarify the functions of IH-NK cells during chronic HCV infection.

In general, due to difficulties to obtain fresh liver biopsies, most of the previous analyses were performed either in small cohorts or on frozen liver biopsies. Unfortunately, these approaches may lead to biased results or misinterpreted data because of i) the wide heterogeneity among limited number of patients, or ii) the possibility of unspecific activations and modifications as consequences of defrost tissue.

The aim of this study was to determine the IH-NK cell functions immediately after liver biopsies and to clarify if the functions of IH-NK cells from HCV-infected patients are impaired or not. We investigated the capacity of fresh *ex vivo* IH-NK cells to secrete anti-viral cytokines and to exert cytotoxic functions. In parallel, we performed similar experiments using the IH-NK cells from Non-Alcoholic Steato-Hepatitis (NASH) individuals with a NAS score ≥3 (non-infected controls) [16], [17] to clarify how the HCV itself affects the threshold of degranulation activity of IH-NK cells during viral infection. In addition, we analyzed the ability of IH-NK cells to be activated after the addition of appropriate cytokine or specific target cell stimulation. These data are described herein in the context of clinical parameters of infected patients. Our study demonstrates that IH-NK cells in HCV-infected patients are potentially functional and that their functions depend on the microenvironment of liver.

## Results

### Analysis of intracellular IFN-γ production in IH-NK cells from HCV-infected and NASH patients

**Figure 1:**
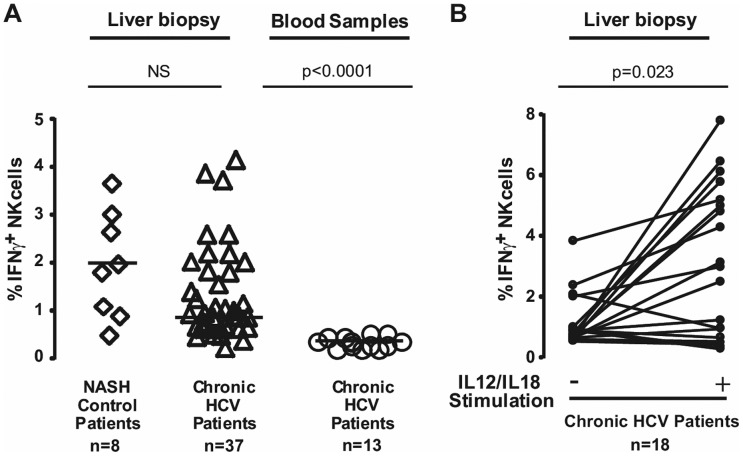
IFN-γ production by NK cells from chronic HCV-infected patients and NASH individuals. **A**) Flow cytometric analysis of spontaneous intracellular IFN-γ production by IH-NK cells, from NASH individuals (n = 8) and HCV-infected patients (n = 37) and by peripheral NK cells from HCV-infected patients (n = 13). **B**) Analysis of IFN-γ-producing IH-NK cells from HCV patients (n = 18) −/+ 12 h of IL12 and IL18 stimulation. Each symbol represented a patient and median values are indicated by dark lines.

NK cells from fresh liver biopsies were analyzed by flow cytometry (Figure S1). Among IH- CD45^+^ population, NK cells were identified by CD56^+^CD3^−^ cellular expression and intracellular IFN-γ production was directly determined. The spontaneous production of IFN-γ cytokine by IH-NK cells was quantified only when liver biopsy contains more than 1500 IH-NK cells. In liver biopsies from HCV-infected individuals (n = 37), the mean frequency of IH-NK cells was 16.1±8.6% and frequencies of IFN-γ^+^IH-NK cells varied from 0.3% to 4.1% (Figure 1A). We also monitored IFN-γ production from peripheral lymphocytes in paired liver biopsies and blood samples (n = 13). However, the frequency of IFN-γ^+^NK cells in blood samples was close to 0.3%. In liver biopsies from NASH individuals (n = 8), the mean frequency of IH-NK cells was 20.1±9.4% and frequencies of IFN-γ producing IH-NK cells varied from 0% to 3.6%. These values were not significantly different from results of IFN-γ production obtained in HCV-infected patients (Figure 1A).

Next, we performed a stimulation assay by adding IL12 and IL18 cytokines to determine if the production of IFN-γ by IH-NK cells from HCV patients could be further activated. To optimize this method, we quantified the amount of IFN-γ released into supernatants of IH-NK cells stimulated with IL12/IL18 cytokines using CBA assays. As shown in Figure S2, IFN-γ was first detectable after 10 h post stimulation. This information allowed us to determine the shortest period of stimulation to precisely study initial population of IH-NK cells at the time of biopsy, i.e. before the expansion of cells, and to avoid the loss of NK cells with time. Thus, we stimulated cytokines over a period of 12 h. Under these conditions, IFN-γ-producing IH-NK cells were detected in 90% of HCV-infected patients (range from 0.3% to 7.8%, n = 18) and the number of IFN-γ^+^IH-NK cells after stimulation was significantly increased (∼ 3.75 fold, p = 0.023) (Figure 1B).

Altogether, these results indicate that at baseline, the frequency of IFN-γ producing IH-NK cells in HCV-infected patients was low, similar to non-infected individuals (Figure 1A) and independent of HCV viral genotypes (Figure S3A). However, the frequency of IFN-γ^+^IH-NK cells can be enhanced by a short exposure of appropriate exogenous stimuli, indicating that a significant number of IH-NK cells are functional in HCV-infected patients and can be recruited after stimulation.

### Analysis of degranulation activity of IH-NK cells from HCV-infected patients

**Figure 2:**
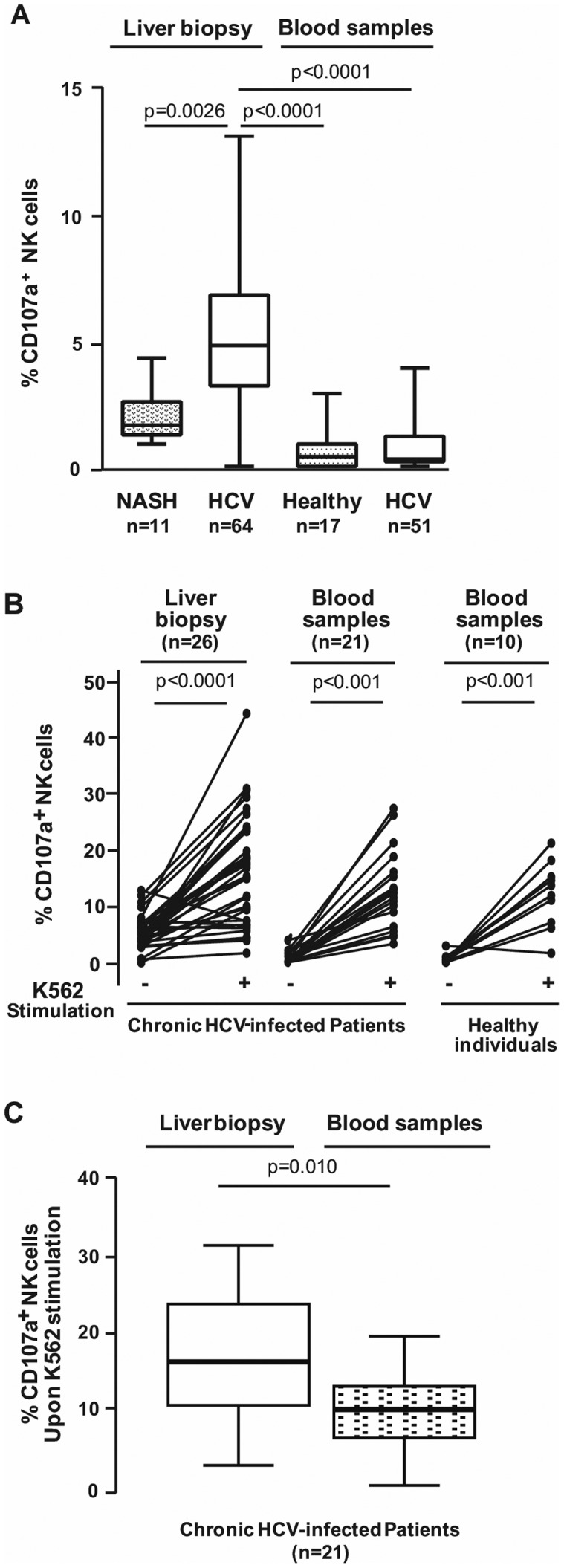
Degranulation activity of NK cells from NASH patients, chronic HCV-infected patients and healthy individuals. **A**) Flow cytometric analysis of CD107a expression by IH-NK cells from NASH individuals (n = 11) and HCV-infected patients (n = 64) directly after the recovery of liver biopsies and by circulating NK cells from healthy donors (n = 17) and HCV-infected patients (n = 51). **B**) Frequencies of degranulating NK cells −/+ 3 h of K562 target cells activation in the liver and the PBMC from HCV-infected patients (n = 36 and n = 21, respectively) and in the blood from healthy donors (n = 10). Each symbol represented a patient. **C**) Simulated degranulation activity of NK cells in the liver and in the blood from HCV-infected patients (n = 21).

To further investigate the cytolytic properties of IH-NK cells, we determined the CD107a expression, which is considered as a marker of degranulation [12], [14], [15]. In 64 HCV-infected samples, IH-NK cells exhibited a spontaneous degranulation activity that varied from 0.1 to 11.2% and was independent of viral genotypes (Figure S3B). The number of CD107a^+^IH-NK cells in HCV-infected patients was significantly higher compared to the 11 non-infected NASH individuals (median 4.8% versus 1.7% respectively, p = 0.0026) as shown in Figure 2A.

When paired liver biopsies and blood samples were available (n = 51), we also monitored degranulation activity from peripheral blood mononuclear cells (PBMC). Very few NK cells were engaged in the degranulation process in blood samples of HCV-infected individuals as well as in the blood (n = 17) of healthy donors (0.3% and 0.4% respectively) (Figure 2A), meaning that the degranulation process of NK cells occurs mainly in the liver organ. It has to be noticed that in this study, degranulation activity monitored via CD107a^+^ expression was assessed at a given time without the use of drugs such as monensin, an inhibitor of cell trafficking. Therefore, our results likely reflect the existence of a permanent “pool” of degranulating IH-NK cells.

We next asked whether the cytolytic activity of IH-NK cells within HCV-infected liver was already maximal or if it could be further increased through stimulation. To answer this question, suspensions of liver NK cells (n = 36) were divided in two parts, and immune cells of one part were incubated during 3 hours with specific K562 target cells deficient in CMH-I (HLA-E) molecule. Upon stimulation, we observed a threefold increase of CD107^+^IH-NK cells (5.4% *vs* 16.4%, p<0.0001) (Figure 2B). Similar increases were observed in peripheral CD107a^+^NK cells from HCV-infected patients (0.2% vs 11.4%, p<0.0001) and from healthy individuals (0.3% vs 12.6%, p<0.0001). Interestingly, the increase of 21 paired CD107a^+^NK cells upon K562 stimulation was statistically higher in the liver than in the blood of HCV-infected patients (17.8% *vs* 11.4%, p = 0.010) (Figure 2C). Taken together, these results indicate that the frequency of cytotoxic IH-NK cells of HCV-infected patients may be further enhanced upon stimulation with specific target cells.

### Analysis of intracellular content of cytotoxic granules in IH-NK cells from HCV-infected patients

**Figure 3:**
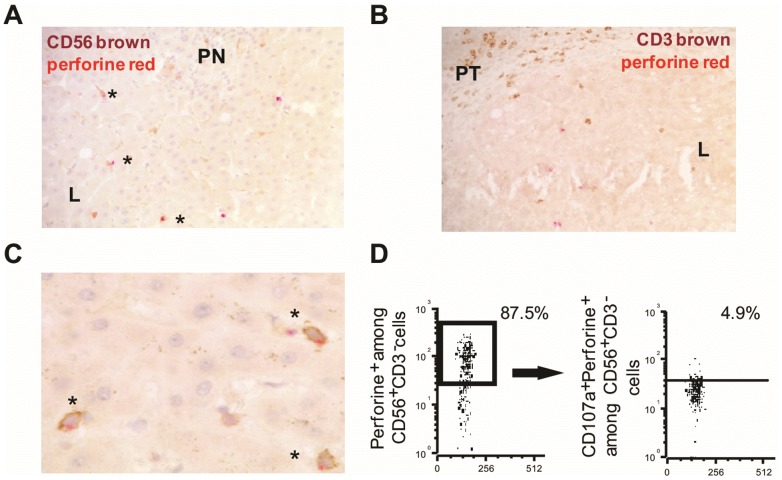
Immunohistochemical detection of perforin^+^CD56^+^ and perforin^+^CD3^+^ IH cells from HCV-infected patients and flow cytometric analysis. Representative pictures from immunohistochemical staining of HCV-infected liver (n = 4) with double perforin/CD56 labeling (A) showing perforin^+^CD56^+^ cells characterized by brown CD56^+^ staining and red cytoplasmic perforin^+^ staining in HCV-infected liver with septal fibrosis and low A1 Metavir activity (magnification ×20). Many double positive CD56^+^perforin^+^ cells (asterisk) were detected, mainly localized in lobules (L), far away from piecemeal necrosis (PN). B) Detection of brown CD3^+^ cells and red perforin^+^ cells in HCV-infected liver with septal fibrosis. Rare double positive CD3^+^perforin^+^ cells were detected in lobules. C) High magnification (x40) showing that perforin granules were polarized at the apical pole of IH-CD56^+^ cells. D) Flow cytometric analysis depicting perforin^+^cells gated on CD56^+^CD3^−^ cells and CD107a^+^ gated on perforin^+^NK cells.

As already mentioned in our previous study [11], intracellular content analysis of IH-NK cells indicated that more than 60% of these cells were loaded with perforin granules whereas only on average of 10% of IH-NKT lymphocytes (CD3^+^CD56^+^cells) were perforin positive. However, no information was available on the localization of immunopositive perforin^+^cells in liver tissue. To investigate this point, we performed double immunohistochemical analyses using anti-CD3 or anti-CD56 combined with anti-perforin antibodies on liver tissue sections on four HCV-infected patients. We observed that double CD56^+^perforin^+^cells were much more frequent (Figure 3A-3B) than double CD3^+^perforin^+^cells. This finding strengthens the fact that most of CD56^+^cells, which contained intra-cytoplasmic perforin^+^ granules, were NK cells. Interestingly, we found that double positive CD56^+^perforin^+^cells were detected mainly in lobular areas far away from parcellar necrosis, whereas CD3^+^perforin^+^cells were detected in fibrotic portal tracts. In addition, as depicted in Figure 3C, perforin granules were observed to be polarized at the apical pole of IH-CD56^+^ cells, indicating that these cells were probably engaged in the specific target cell lysis through degranulation process. Finally, we checked by flow cytometric analysis using a combination of anti-CD3, anti-CD56, anti-perforin, and anti-CD107a antibodies, the presence of CD107a^+^perforine^+^IH-NK cells in the fresh biopsy of 13 HCV-infected patients. We detected CD107a^+^perforin^+^IH-NK cells with a frequency varying from 0.3% to 15.7% among the perforin^+^IH-NK cells (median of 5.6%). This data underline the fact that IH-NK cells of HCV-infected patients exerted a cytolytic activity.

### Relationship between the number of IH-NK cells producing IFN-γ and clinical parameters in HCV-infected patients

**Figure 4:**
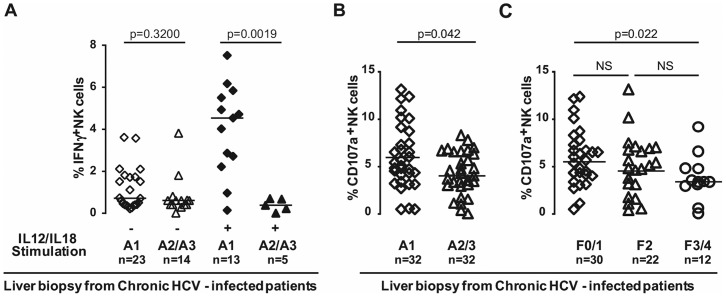
Relationship between intra-hepatic NK cells functions and clinical parameters of HCV-infected patients. **A**) The number of IFN-γ^+^IH-NK cells with and without IL12/IL18 stimulation in patients stratified according to stage of Metavir activity score (A1; A2/A3). The spontaneous degranulation activity in patients with different Metavir activity grade (**B**) and in patients distributed according to the severity of fibrosis (**C**). Each patient is represented by a symbol and median values are indicated by dark lines.

Then, we investigated the relationship between the frequencies of IH-NK cells producing IFN-γ and the intensity of necro-inflammatory lesions of HCV-infected patients. The liver samples of 37 HCV-infected patients were stratified into two groups based on Metavir activity score: A1 and A2/A3. We observed that in not-stimulated cells, the frequency of IFN-γ-producing IH-NK cells is similar for patients with Metavir activity A1 compared to A2/A3. Interestingly, after stimulation, the frequency of IFN-γ-producing IH-NK cells in patients with A1 Metavir activity (n = 13) was 5 fold higher and statistically different (p = 0.0019) compared to patients with A2/3 Metavir activity (n = 5) (Figure 4A).

### Relationship between degranulation activity of NK cells and clinical parameters in HCV-infected patients

We next studied the correlation between the frequency of CD107a^+^IH-NK cells and the clinical parameters. We found that the number of CD107a+ IH-NK cells was slightly higher in patients with A1 compared to A2/A3 Metavir Activity (p = 0,042) (Figure 4B). Metavir fibrosis stage inversely correlates with the degranulation activity of IH-NK cells (Figure 4C); this is supported by the observation of 5.7% of IH-NK cells from HCV-infected patients were involved in the degranulation process at F0/1 stage while only 3.4% of CD107a^+^IH-NK cells were detected at F3/4 stage (p = 0.022). These results suggest that IH-NK cells from HCV-infected patients display a sustaining spontaneous degranulation activity that is partially reduced with the progression of liver inflammation and fibrosis.

## Discussion

Recent observations have shown that NK cell functions in mice are strongly influenced by their environment and that these cells display a greater capacity to adapt to their local context than was previously expected [18]. In addition, the activation of NK cells is dependent on the engagement of activating and inhibitory receptors that determines the reactivity of NK cells [18]. The functions of peripheral blood NK cells in HCV-infected patients have been extensively studied in the last decades and are still debated. During acute HCV infection, the cytotoxic functions of peripheral NK cells and their production of IFN-γ, that can directly inhibit viral replication and prime the adaptive immune response, are higher compared to healthy individuals [19]. In contrast, during chronic HCV infection, the production of IFN-γ decreases and contributes to viral persistence (see reviews [20], [21]).

A large number of studies have focused on the functions of peripheral NK cells however analyses of IH-NK cells are very limited. In addition, most of the available data were obtained on frozen liver tissues and cells cultured with cytokines (i.e. IL2), therefore modifying the experimental environment of NK cells present in infected liver can lead to biased conclusions about their functions. Some works provide evidence that IH-NK cell degranulation activity is higher compared to peripheral NK cells [12], [22] and can be further activated by IFN-α antiviral therapy of HCV infection [15]. On the other hand, another study suggests that IH-NK cell cytotoxic function is impaired in patients with chronic HCV infection while peripheral NK cells are activated [14].

Here, *ex-vivo* analysis of fresh liver biopsies and blood samples indicates that IH-NK cells have higher degranulation activity and secrete more IFN-γ than peripheral NK cells in HCV-infected patients. Moreover, our data provide evidence that IH-NK cells from HCV-infected patients display higher cytotoxic activity but produce similar amount of IFN-γ as IH-NK cells from non-infected patients. In addition, immunohistochemical approaches show that some IH-NK cells of HCV infected individuals display perforin-containing granules polarized at the apical pole of the cells and that some perforin^+^ IH-NK cells are CD107^+^, indicating they are engaged in a degranulation process. By focusing our analysis on the functional responsiveness of IH-NK cells in chronically HCV-infected patients rather than a phenotypic analysis of their activating and inhibitor receptors, we observe that only a limited number of NK cells are reactive whereas it has been previously reported that the majority of those cells express activating receptors such as NKG2D and NKp46 [7], [15], [22]. This limited pool of active IH-NK cells in chronic HCV infection might be the result of a failure of the signalling network such as the decrease of IFN-α production that is required to activate immune cells including NK cells [20], [21], [23]–[25]. Another reason may be that HCV directly inhibits immune cells by contact with HCV-infected hepatocytes as demonstrated *in vitro* [26]. Furthermore, as described in other viral infections, one may propose that IH-NK cells reduce the number of infected antigen presenting cells, thereby facilitating the presence of the virus in the liver tissue [27] or that the NKp46^+^ IH-NK cells tune their threshold of responsiveness and become tolerant that is similar to the phenotypic behaviour described in neutropenic mice [28]. With respect to the fact that the activity of NK cells is critically affected by a variety of other factors, including dendritic cells, monocytes or CD4^+^ T cells [4], it is difficult to assess precisely what factors actually limit NK cell activity in the liver of HCV infected patients. However, we show that the frequencies of NK cells producing IFN-γ or degranulating increase after in vitro stimulation by either IL-12 and IL-18 cytokines or by target cells. This supports the fact that some NK cells, even though they remain capable of reactivation, are partially inhibited because either they are not triggered by the right signals to fully exert their functions or they modulate themselves the level of their reactivity in the liver tissue. Furthermore, the frequency of IFN-γ producing NK cells and to a lesser extent the frequency of degranulating NK cells, decrease as the liver inflammation status increases (Metavir Activity Grade and Fibrosis Stage). Therefore, as the inflammatory status remains low, a fraction of NK cells exert their functions, whereas these functions are altered when the inflammatory status of the liver increases. Two hypotheses may account for this observation: either part of NK cells become non reactive because the inflammatory context prevents their activation, or NK cells adapt themselves to their environment by reducing the cytokines production and the release of cytotoxic granules in the aim of limiting tissue damage. We privilege this latter hypothesis that converges with conclusions of animal model studies showing that NK cells reactivity is tuned by the microenvironment and depends on the time of exposure to this environment [28]. In addition, it is also possible that a decline in NK cell activity itself further favours development of liver fibrosis. This is in agreement with the assumption that IH-NK cells during long-lasting chronic infection are hyporesponsible and enable to ensure their role in immunosurveillance, as suggested by Mondelli and others [6], [29]. Taken together, IH-NK cells, although activated, are unable to clear the HCV-infection on their own when the infection is chronic, which seems to be a general feature of IH-NK cells rather than the result of viral escape strategy.

In our study, we took the advantage of combining at the same time the reactivity of IH-NK cells with the evaluation/diagnostic of histological damage. Thus, our observations indicated that after stimulation, IH-NK cells were producing IFN-γ at early phases of the liver inflammation disease. This may explain the controversy in current literature that was focusing on IH-NK cell functions during chronic HCV infection (see review [20], [21]). More importantly, this finding should be taken into consideration for novel therapeutic strategies that intend to involve direct activation of IH-NK cells or to block IH-NK cell inhibitory receptors in order to enhance the induction of anti-viral cytotoxic activities [24]. Our results also suggest that for successful management of chronic-HCV infection treatment is necessary to focus on the first stage of the liver disease when the tissue damage is not yet significantly developed.

Overall, our study provides clear evidence that some IH-NK cells in HCV-infected patients exert degranulation activity and produce IFN-γ and can be further activated, opening new issues in the follow up of the patients.

## Materials and Methods

### Patients

One hundred twenty patients included in this study were prospectively selected prior any treatment and separated into 2 groups according to the aetiology of chronic liver disease: Group 1, including 101 patients, chronic HCV (Department of Gastroenterology and Hepatology, Grenoble University Hospital). All patients were anti-HCV antibody positive (ELISA 3, Ortho Diagnostic Systems, Raritan, NJ, USA) and serum HCV-RNA positive by RT-qPCR (Amplicor HCV, Roche Diagnostic Systems, Meylan, France). HCV genotype was determined by Inno-Lipa technology (Innogenetics, Zwijnaarde, Belgium).; Group 2, including 19 patients with histologically proven Non Alcoholic Fatty Liver Disease (NAFLD), considered as non-infected control group with NAFLD Activity Score (NAS) ≥ 3, corresponding to borderline NASH with low necroinflammatory activity (NAS 3 or 4) and definite NASH (NAS≥5). Histological examination was assessed by experienced liver pathologist according to Metavir scoring system for HCV patients and according to Brunt's [16] and Kleiner's [17] scoring system for NASH patients. However, necroinflammatory activity and fibrosis stage of NASH patients were also expressed according Metavir classification, allowing the comparison between HCV and NASH patients. Patients were negative for HIV and HBV infection, without biochemical markers of autoimmune hepatitis, and their alcohol consumption was lower than 30 g/day in male and 20 g/day in women. The main characteristics of all patients are described in Table S1.

Liver biopsies were divided into two parts: one part for histological examination and the other part for immunological analyses [30]. Patients were divided in two cohorts, the first one for IFN-γ-study (37 HCV-infected biopsies and 8 NASH biopsies), and the second cohort for CD107-study (64 HCV-infected biopsies and 11 NASH biopsies). When the length of HCV-infected biopsy was more than 1 cm, the liver specimen was divided in two parts to perform analyses at baseline and after stimulation. Paired blood and liver simple were obtained in 13 HCV-infected patients for IFN-γ study and 51 HCV-infected patients for CD107 study, among this blood samples 21 were stimulated (Figure S4).

PBMC from 17 healthy individuals were collected from EFS (Etablissement Français du Sang). The study was performed in accordance with the Declaration of Helsinki and French legislation, and received approval of the Grenoble University Hospital ethical committee (03/APTF/1). All study participants provided written informed consent.

### Flow cytometric analysis

Immediately after the liver biopsy, cells were recovered by mechanical disruption, and immunostained for flow cytometric analysis. The leucocytes of blood sample were extracted with Filcool and the same protocol for immunostaining as for liver samples was used. Immuno-phenotyping was performed on fresh PBMC and liver samples with the following anti-human antibodies: CD3-PE-Cy7, CD56-PE from Beckman Coulter, CD56-FITC, IFN-γ-PE, Perforin-FITC and CD107a-APC from Becton-Dickinson Biosciences, CD3-APC-Cy7, CD45-APC-Cy7, uncoupled CD107a and CD107a Pacific Blue from Biolegend analyses were done as previously described [7], [30]. Data were acquired on BD LSRII flow cytometer (BD Biosciences) and analyzed using FCS Express V3 software.

### IFN-γ production following IL12/IL18 stimulation

Liver biopsies from 18 out of 37 HCV-infected patients were mechanically disrupted and cultured in RPMI medium in a 5% CO_2_ incubator at 37°C with or without IL12 (20 ng/mL; R&D Systems) and IL18 (80 ng/mL; MBL Clinisciences) as described [31]. Brefeldin A (GolgiPlug, Becton Dickinson) was then added to detect intracellular IFN-γ 6 h after initiation of stimulation and culture was maintained for another 6 h before harvesting. Finally, cells were washed and stained prior to flow cytometric analysis. IFN-γ released by liver-infiltrating immune cells was performed on 3 HCV-infected patients with or without incubation with IL12+IL18 over 24 h. Supernatant samples were recovered at 0, 2, 4, 6, 8, 10, 12 and 24 h post stimulation and IFN-γ secretion was measured by Cytometric Bead Array (CBA) assay according manufacturer's instruction and analyzed by flow cytometry.

### Degranulation activity following K562 target cells activation

IH or PBMC immune cells were incubated for 3 h at 37°C with or without K562 target cells (Cell:Target = 1:1). Cells were then harvested, washed and stained for flow cytometric analysis. Degranulation activity was monitored by detection of membranous presence of CD107a marker [12], [14], [15], [32].

### Immunohistochemical study

Double perforin/CD56 and perforin/CD3 immunostainings were performed on consecutive 4 µm thick liver sections (n = 4) from formalin-fixed, paraffin-embedded tissue, as described in [33]. Slides were first immunostained with the anti-perforin Ab2 antibody (Clone 5B10, Neomarkers) and revealed with the N-Histofine kit (Nichirei Biosciences Inc). Fast red was used as the chromogen for perforin immunodetection. Then, antigen retrieval was performed in Tris-citrate buffer for CD56 or citrate buffer for CD3. Slides were incubated with the primary anti-CD56 (clone 1B6, Novocastra) or anti-CD3 (polyclonal rabbit CD3, Dako) antibodies and then incubated with the secondary antibody using the Envision kit. DAB was used as the chromogen for CD56 or CD3 immunodetection. All sections were counterstained with Harris hematoxylin, washed in tap water and mounted in aquamount (Merck). Sur-fixation in glutaraldehyde (0.05% in PBS pH 8.6) followed each incubation with the primary or secondary antibody.

### Statistical methods

Statistical analysis was performed using SPSS 19.0 software (*SPSS Inc., Chicago, IL, USA*) as previously described [33] and included the following statistical tests: Mann-Whitney U test, Pearson and Wilcoxon matched-t test. Two-sided *P* values < 0.05 were considered to be significant.

## Supporting Information

Figure S1 Flow cytometry strategy to investigate intracellular production of IFN-γ and degranulation activity of NK cells. IH lymphocytes were first identified according their FSC and SSC parameters (gate 1) and further gated on their CD45^+^ expression (gate 2). Among the CD45^+^ population, NK cells (CD56^+^CD3^−^) were then analyzed for their intracellular contents of IFN-γ cytokine or perforin and CD107a expression.
(PDF)

Figure S2 Detection of IFN-γ IH-NK cells production from chronic HCV-infected patients after stimulation with IL12/IL18. Monitoring of IFN-γ production was performed on 3 liver biopsies from HCV-infected patients incubated or not with IL12/IL18 over 24 h. Supernatant was recovered at 0, 2, 4, 6, 8, 10, 12 and 24 hours and IFN-γ release was measured by CBA assays, and analyzed by flow cytometry.
(PDF)

Figure S3 Relationship between IH-NK cells functions and HCV viral genotypes. **A**) The number of IFN-γ^+^IH-NK cells and (**B**) degradulation activity in patients stratified according to HCV viral genotype. Each patient is represented by a symbol and median values are indicated by dark lines.
(PDF)

Figure S4 The frequencies of IH-NK cells in studies. NK cells from fresh liver biopsies and from blood samples of HCV-infected patients were analyzed during IFN gamma and CD107 study. The frequencies of IH-NK cells (mean ± SD) were determined.
(PDF)

Table S1 Main characteristics of the patients. Demographic, biochemical and clinical parameters of chronic HCV-infected patients and of NASH individuals studied for production of IFN-γ and CD107a expression. Necroinflammatory activity and fibrosis stage of NASH patients were expressed according Metavir classification, allowing the comparison between HCV and NASH patients.
(PDF)
