# Supplementary figures and images for: Functions of Liver Natural Killer Cells Are Dependent on the Severity of Liver Inflammation and Fibrosis in Chronic Hepatitis C
Source: PLoS One. 2014 Apr 23;9(4):e95614. doi: 10.1371/journal.pone.0095614 (PMC3997478; doi:10.1371/journal.pone.0095614)

Supplementary Figure 1.

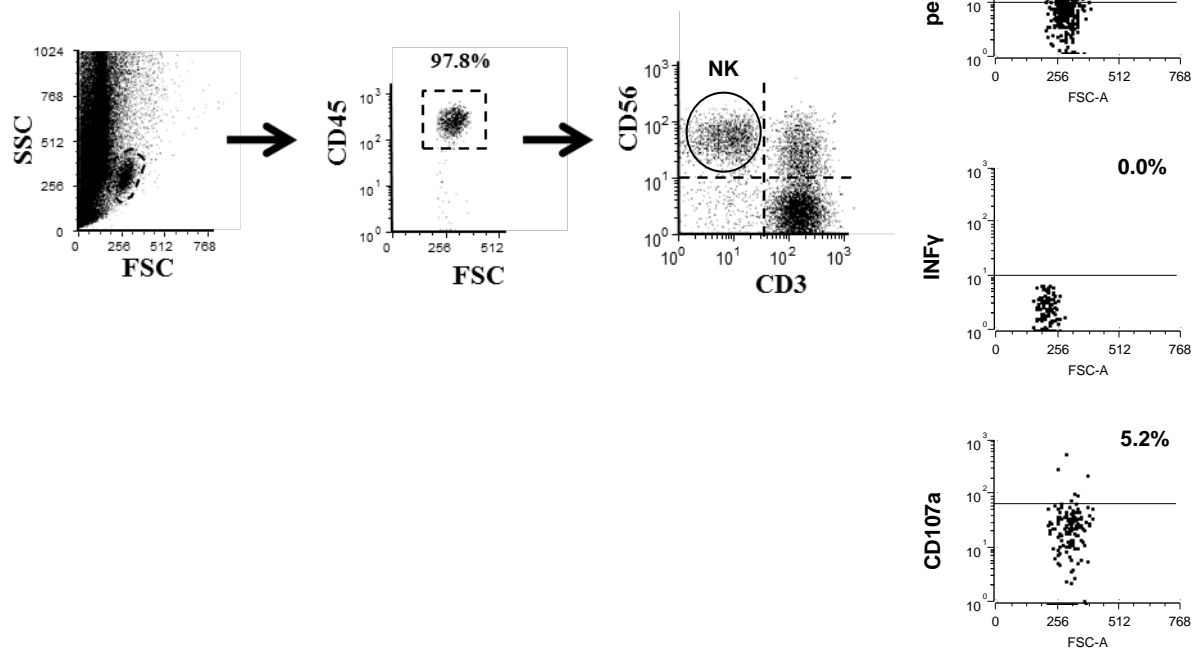

Supplement: Figure S1 — Flow cytometry strategy to investigate intracellular production of IFN-γ and degranulation activity of NK cells. IH lymphocytes were first identified according their FSC and SSC parameters (gate 1) and further gated on their CD45+ expression (gate 2). Among the CD45+ population, NK cells (CD56+CD3−) were then analyzed for their intracellular contents of IFN-γ cytokine or perforin and CD107a expression. (PDF) [file pone.0095614.s001.pdf]

Supplementary Figure 2

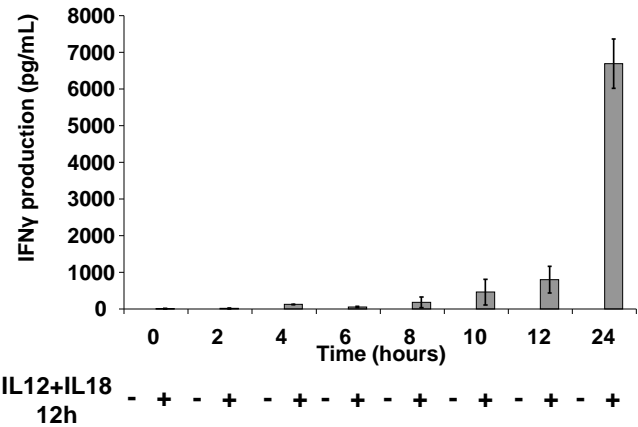

Supplement: Figure S2 — Detection of IFN-γ IH-NK cells production from chronic HCV-infected patients after stimulation with IL12/IL18. Monitoring of IFN-γ production was performed on 3 liver biopsies from HCV-infected patients incubated or not with IL12/IL18 over 24 h. Supernatant was recovered at 0, 2, 4, 6, 8, 10, 12 and 24 hours and IFN-γ release was measured by CBA assays, and analyzed by flow cytometry. (PDF) [file pone.0095614.s002.pdf]

**A**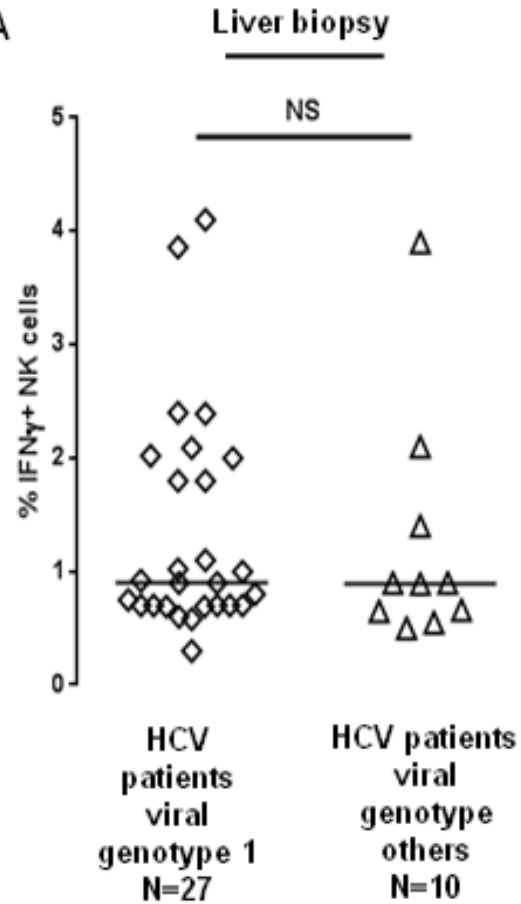**B**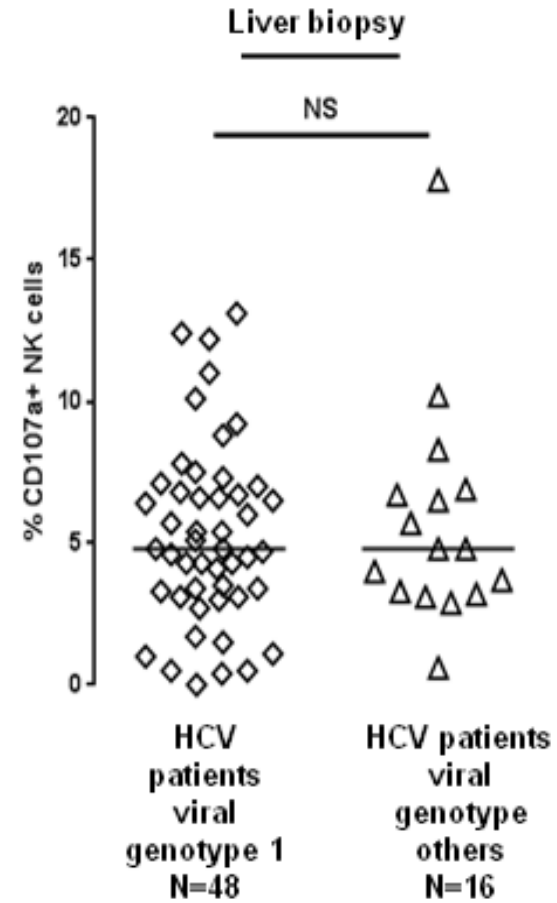

Supplement: Figure S3 — Relationship between IH-NK cells functions and HCV viral genotypes. A) The number of IFN-γ+IH-NK cells and (B) degradulation activity in patients stratified according to HCV viral genotype. Each patient is represented by a symbol and median values are indicated by dark lines. (PDF) [file pone.0095614.s003.pdf]

**A****IFN $\gamma$  study:**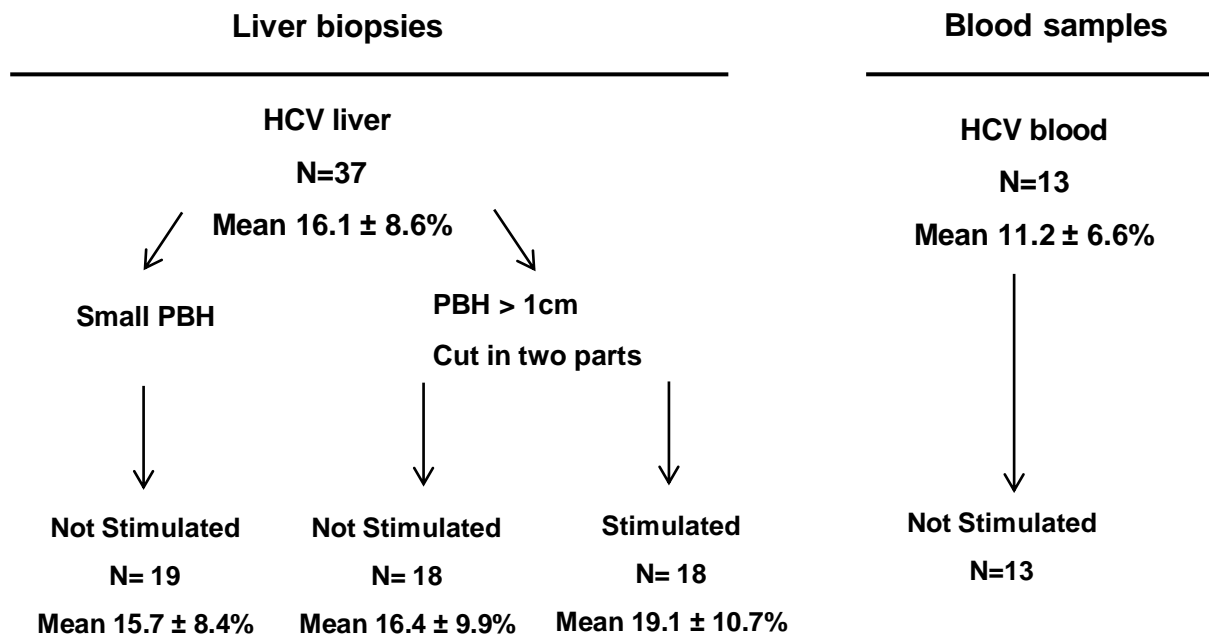**B****CD107 study:**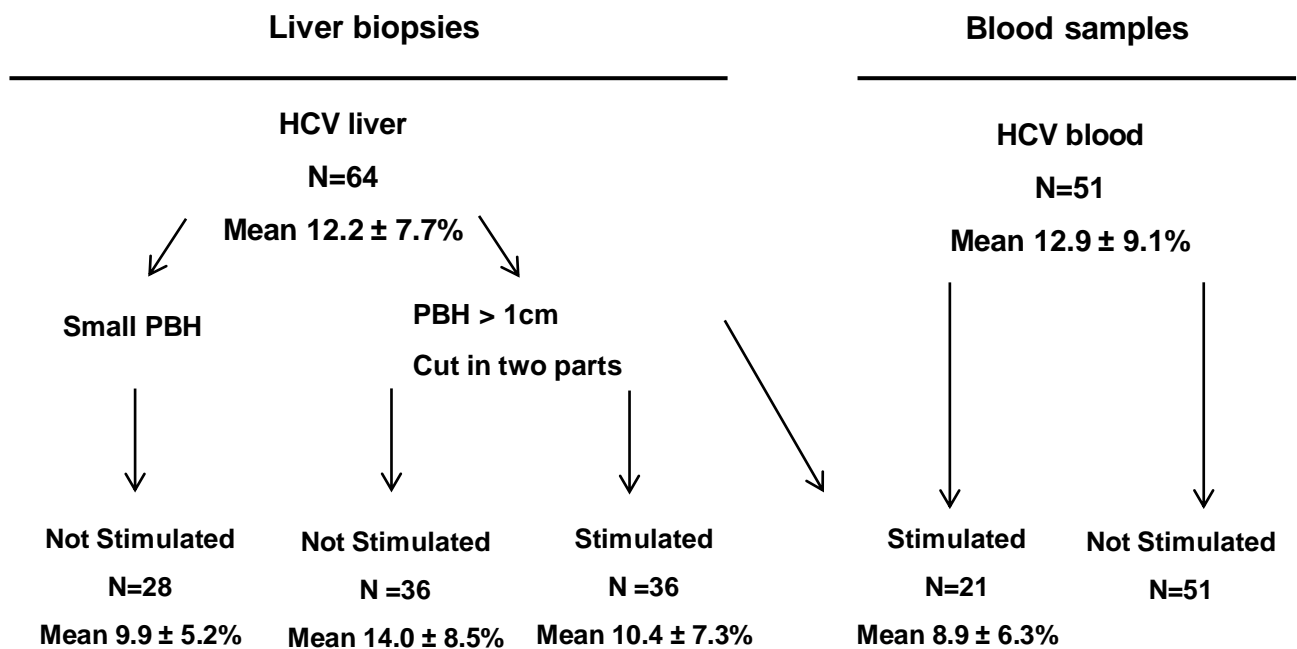

Supplement: Figure S4 — The frequencies of IH-NK cells in studies. NK cells from fresh liver biopsies and from blood samples of HCV-infected patients were analyzed during IFN gamma and CD107 study. The frequencies of IH-NK cells (mean ± SD) were determined. (PDF) [file pone.0095614.s004.pdf]
