# Supplementary material for: Functions of Liver Natural Killer Cells Are Dependent on the Severity of Liver Inflammation and Fibrosis in Chronic Hepatitis C
Source: PLoS One. 2014 Apr 23;9(4):e95614. doi: 10.1371/journal.pone.0095614 (PMC3997478; doi:10.1371/journal.pone.0095614)
Supplement: Table S1 — Main characteristics of the patients. Demographic, biochemical and clinical parameters of chronic HCV-infected patients and of NASH individuals studied for production of IFN-γ and CD107a expression. Necroinflammatory activity and fibrosis stage of NASH patients were expressed according Metavir classification, allowing the comparison between HCV and NASH patients. (PDF) [file pone.0095614.s005.pdf]

Supplementary Table 1.

	HCV-infected patients (Group 1)				NASH individuals (Group 2)			
	**IFN-γ Study**		**CD107a Study**		**IFN-γ Study**		**CD107a Study**	
	N=37		N=64		N=8		N=11	
Male, n (%)	18	49%	37	58%	2	25%	8	72%
Age (y)*	55.2	± 6.7	52.6	± 9.5	65.9	± 9.5	57.3	± 12.6
Plasma Viral load (log IU/mL)*	6.1	± 0.8	5.9	± 0.9	-	-	-	-
Serum ALT (IU/mL)*	84.9	± 34.6	100.1	± 91.8	55.0	± 49.5	81.0	± 117.6
Genotype Viral 1	27	73%	48	75%	NA		NA	
Metavir Activity Score, n (%)								
A0	0	-	0	-	3	37%	6	55%
A1	23	62%	34	53%	3	37%	4	36%
A2/3	14	38%	30	47%	2	25%	1	9%
Metavir Fibrosis score, n (%)								
F0/1	18	49%	30	47%	7	88%	6	55%
F2	15	40%	22	34%	0	-	1	9%
F3/4	4	11%	12	19%	1	12%	4	36%

*average ± standard deviation
